# ToF-SIMS sputter depth profiling of interphases and coatings on lithium metal surfaces

**DOI:** 10.1038/s42004-025-01426-0

**Published:** 2025-02-03

**Authors:** Maximilian Mense, Marlena M. Bela, Sebastian P. Kühn, Isidora Cekic-Laskovic, Markus Börner, Simon Wiemers-Meyer, Martin Winter, Sascha Nowak

**Affiliations:** 1 Münster Electrochemical Energy Technology (MEET), University of Münster, Münster, Germany; 2 Helmholtz-Institute Münster, IMD-4, Forschungszentrum Jülich GmbH, Münster, Germany

**Keywords:** Batteries, Mass spectrometry

## Abstract

Lithium metal as a negative electrode material offers ten times the specific capacity of graphitic electrodes, but its rechargeable operation poses challenges like excessive and continuous interphase formation, high surface area lithium deposits and safety issues. Improving the lithium | electrolyte interface and interphase requires powerful surface analysis techniques, such as ToF-SIMS sputter depth profiling.This study investigates lithium metal sections with an SEI layer by ToF-SIMS using different sputter ions. An optimal sputter ion is chosen based on the measured ToF-SIMS sputter depth profiles and SEM analysis of the surface damage. Further, this method is adapted to lithium metal foil with an intermetallic coating. ToF-SIMS sputter depth profiles in both polarities provide comprehensive insights into the coating structure. Both investigations highlight the value of ToF-SIMS sputter depth profiling in lithium metal battery research and offer guidance for future studies.

## Abstract (web summary)

Optimizing lithium–electrolyte interfaces for Li metal batteries requires the use of powerful surface analysis techniques. Here, the authors demonstrate the value of ToF-SIMS sputter depth profiling in lithium metal battery research and offer guidance for future studies.

## Introduction

Climate change is one of the most urgent challenges humanity is facing - as a result, there is a global push towards renewable energy sources and sustainable mobility. Stationary and mobile energy storage solutions are inevitable for this change, as they help to stabilize the power grid and enable electric vehicles^1,2^. In both application cases, lithium ion batteries (LIB) are state-of-the-art technology, though the currently used electrode materials are limiting in terms of specific energy and energy density. Next-generation batteries have the potential to overcome these limitations by implementation of innovative materials. A promising strategy is the use of lithium metal negative electrodes due to their high theoretical specific capacity (3860 mAh⋅g^−1^) and low standard reduction potential (−3.04 V vs. standard hydrogen electrode) compared to all metals^3,4^. However, lithium metal electrodes are prone to safety issues and performance degradation. This is related to the plating morphology of metallic lithium, which can exhibit high surface area lithium, for instance in the form of dendrites^5–7^. Organic liquid electrolytes are electrochemically instable versus lithium metal, leading to reductive electrolyte decomposition accompanied by a loss of electrochemically active lithium and a rise of internal resistance. Fortunately, a solid electrolyte interphase (SEI) is formed from hardly soluble decomposition products, which, in the ideal case stops the ongoing decomposition^8–10^. The structure of the formed SEI is complex and composed of multiple organic and inorganic components, depending on the electrolyte used. A common model in literature is the “polyhetero-microphase” SEI proposed by Peled et al.^11^. In this model, the SEI consists of a mosaic of micro-phases/domains which deposit on the lithium metal surface. Within this mosaic, the outer areas are reported to be rich in organic compounds (e.g., semicarbonates, polymers), while the inner areas, close to the lithium metal, are rich in inorganic lithium compounds (e.g., lithium oxide, lithium carbonate, lithium fluoride)^12–14^.

Current research focuses on improving the lithium deposition properties by modification of the electrode surface and development of new electrolyte formulations^6,15–17^. These strategies aim for an increased control over the morphology of the plated lithium and for decreasing parasitic SEI-formation reactions^18,19^. In order to address these research approaches, a deeper understanding of fundamental reactions occurring at the lithium metal surface and of the SEI chemistry is required. Surface analysis techniques play a crucial role in these investigations. However, the analysis of lithium metal and its SEI is accompanied with challenges due to low sensitivity for lithium in X-ray-based techniques and its high reactivity with atmospheric components. One technique that can handle these challenges is time of flight—secondary ion mass spectrometry (ToF-SIMS).

ToF-SIMS can provide comprehensive mass spectrometric information on chemical surface composition and the in-depth structure of the material’s surface. This technique is able to gain elemental and molecular information on the top layers of the sample by the bombardment of the sample surface with primary ions (PI) and the analysis of resulting secondary ions (SI). By implementation of a secondary sputter ion source, a continuous removal of material enables the analysis of initially covered layers^20–23^. For ToF-SIMS analysis of reactive samples such as lithium metal, the use of an Ar-filled transfer vessel provides an inert atmosphere during sample transfer to the instrument, this ensures an unimpaired sample surface for analysis.

For ToF-SIMS sputter depth profiling, the choice of sputter ion influences both the sputter properties (e.g., sputter yield) and the mass spectrometric information gained by SIMS analysis. In general, a distinction can be made between monatomic and polyatomic sputter ions. Monoatomic sputter (e.g., Cs^+^, Ar^+^, O_2_^+^) ions have a higher energy per atom compared to polyatomic sputter ions (e.g., Ar_1500_^+^ and larger). Depending on the acceleration voltage used, the energy per ion is between 250 eV and 2 keV (may differ for other instruments). In comparison, the energy per polyatomic sputter ions is typically at 5–10 keV (may differ for other instruments or polyatomic ions) resulting in an energy per atom in the low eV range, e.g., 3.33 eV for 5 keV Ar_1500_^+^ sputter ions. As a result, the sputter ion-induced fragmentation of analytes (e.g., molecular species) is more pronounced for monoatomic sputter ions than for polyatomic sputter ions. Argon cluster ions are commonly used in ToF-SIMS sputter depth profiling of organic or polymeric samples, due to the preservation of molecular information^24–26^. Although the cluster ions are mostly used for polymer investigations, they were already applied to lithium metal investigations due to their inert characteristics^27,28^. Gas cluster ion sources, as employed in this study, are technically limited to the preparation of a cluster ion distribution, encompassing ions of varying sizes in comparison to the main cluster size. In this work, the cluster size (e.g., Ar_1500_^+^) is stated by the main cluster size of this distribution, which was targeted during the daily instrumental tuning process, but includes a distribution of cluster ion sizes. A widely used monoatomic sputter ion for ToF-SIMS sputter depth profiling is Cs^+^^29–33^. In negative SIMS polarity, Cs^+^ sputter ions increase SI yield, as a monolayer of cesium is deposited on the sample surface, donating electrons to the surface, thus leading to a reduction of the surface^22,23,34,35^. A comparable characteristic is known for O_2_^+^ sputter ions, leading to the oxidation of the sample surface and an increased SI yield in positive SIMS polarity. In addition to these SI yield-enhancing effects, the analysis of sputter-induced Cs-cluster SI (*M*Cs^+^ and *M*Cs^2+^; *M* analyte ion) is reported to reduce matrix effects during ionization and enable semi-quantitative elemental depth profiling for inorganic multi-layer systems^23,34–38^. Another common monoatomic sputter ion is Ar^+^, which is used in X-ray photoelectron spectroscopy as well as ToF-SIMS sputter depth profiling^14,39,40^. These Ar^+^ ions miss the SI yield enhancing characteristic in contrast to Cs^+^ and O_2_^+^ ions. However, the use of Ar^+^ ions allows for SIMS in positive and negative modes without reduction/oxidation of the surface and for an overlay of depth profiles in both SIMS polarities under identical sputter conditions.

The objective of this study is to identify a well-suited sputter ion species and give corresponding ToF-SIMS parameters for an optimized analysis of SEI on lithium metal. Therefore, a selection of sputter ions (Ar_1500_^+^, Cs^+^ and Ar^+^) is compared regarding their sputter characteristics and the measured ToF-SIMS depth profiles of SEI on lithium metal samples. Moreover, the sputter parameters are adapted for the analysis of thicker layers on lithium metal, such as artificial protective layers. The results advance ToF-SIMS sputter depth profiling as a tool for comprehensive surface analysis of lithium metal electrodes.

## Results and discussion

### Comparison of sputter ion species and parameters for thin-layer depth profiling

The first part of this study focuses on the comparison of sputter ions for the SEI analysis on lithium metal. The objective was to balance sufficient depth resolution and measurement time while preserving surface integrity. A lithium metal section covered with SEI was measured by ToF-SIMS depth profiling using three different sputter ions (Ar_1500_^+^, Cs^+^, and Ar^+^). The section was prepared by a liquid–solid reaction, when cutting a lithium metal rod immersed in a commonly-used organic carbonate-based electrolyte. The contact of bare lithium metal and the electrolyte resulted in the formation of a SEI layer on the cutting surface^41^.

**Fig. 1:**
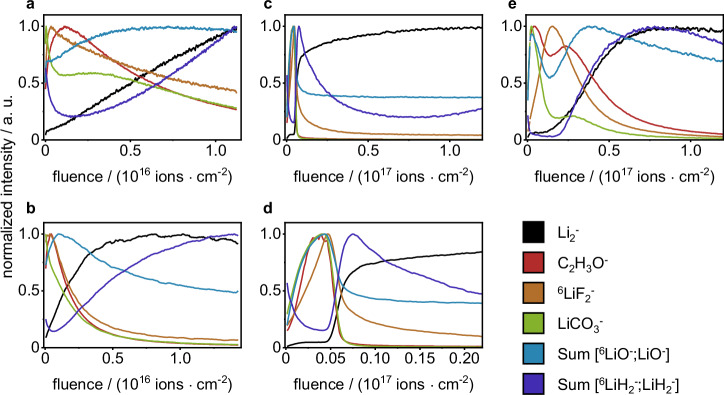
Comparison of ToF-SIMS sputter depth profiles utilizing different sputter ion species. SI intensities are normalized to the respective maximum. The given fluence corresponds to the sputter ion fluence. The profiles correspond to **a** 5 keV Ar_1500_^+^ 500 pA, **b** 10 keV Ar_1500_^+^ 5 nA, **c** 500 eV Cs^+^ 20 nA, **d** zoom-in of (**c**), **e** 500 eV Ar^+^ 20 nA.

**Fig. 2:**
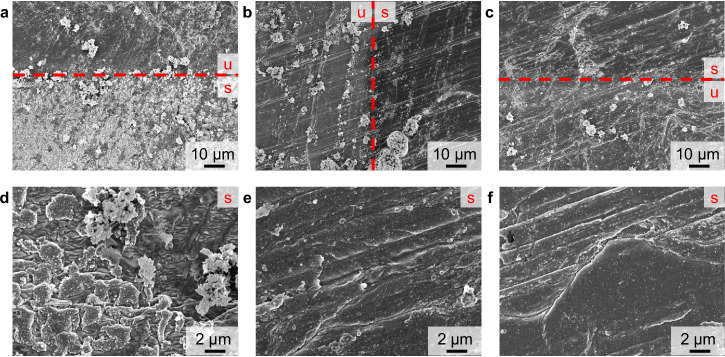
SEM images of the lithium metal surface after sputter ion bombardment. The unsputtered surface is marked by a red ‘u’ and the sputtered area by a red ‘s’, the red dashed line indicates the transient region. In **a**–**c**, the surface for 10 keV Ar_1500_^+^, Cs^+^, and Ar^+^ at 1k magnification are given, **d**-**f** depict the surfaces after sputter depth profiling for 10 keV Ar_1500_^+^, Cs^+^ and Ar^+^ at 5k magnification.

At first, ToF-SIMS depth profiling was performed using Ar_1500_^+^ sputter ions at an acceleration voltage of 5 keV and a sputter ion current of 500 pA, as these parameters were published for the analysis of thin layer depth profiling on lithium metal^27^. Sputtering with these sputter parameters, did not reach the bulk material in a reasonable time (stopped at sputter ion fluence of 1.1 × 10^16 ^ions⋅cm^−2^ equals >45 min measurement time), as the bulk-attributed SI LiH_2_^−^ and Li_2_^−^ did not reach a plateau intensity, but increased continuously (Fig. 1a). Likely this continuous increase of intensity is due to an ongoing removal of the SEI layer, but no complete removal throughout the measurement. The SI LiH_2_^−^ was chosen as a SI of lithium hydride that is reported to be present on the lithium metal surface^27^. However, further studies are needed to clearly verify the presence of lithium hydride. Additionally, the SI Li_2_^−^ was chosen as indication for metallic lithium. Subsequent measurements in this study showed that both SI (LiH_2_^−^ and Li_2_^−^) were present in the depth of the surface layer of lithium metal and were not followed by other relevant SI for bulk lithium metal. (e.g., Fig. 1c). In the measured 5 keV Ar_1500_^+^ ToF-SIMS depth profile, the maximum intensity for LiCO_3_^−^ was detected at the very top layer, followed by the maximum intensity for ^6^LiF_2_^−^. The depth profile of LiCO_3_^−^ featured a decrease with an intensity shoulder, in contrast to this, ^6^LiF_2_^−^ intensity decreased at a constant rate. Whilst LiCO_3_^−^ and ^6^LiF_2_^−^ intensities decreased, there was a maximum intensity reached for C_2_H_3_O^−^, which subsequently also decreased at a steady rate. The LiO^−^ intensity was found to constantly increase, reaching its maximum intensity at a higher sputter ion fluence compared to C_2_H_3_O^−^. Throughout the rest of the measurement, the LiO^−^ intensity decreased at a very minor rate. These observations are partly in accordance with common SEI models for lithium metal, postulating a layer of organic decomposition products (C_2_H_3_O^−^ and LiCO_3_^−^) close to the electrolyte, followed by inorganic products as lithium fluoride (^6^LiF_2_^−^), lithium carbonate (LiCO_3_^−^) and lithium oxide (LiO^−^) near to the lithium metal surface^28^. However, in this SEI model it is not expected that lithium carbonate (LiCO_3_^−^) is only present at the very top, as well as organic species (C_2_H_3_O^−^) are not expected to be present underneath the lithium fluoride (^6^LiF_2_^−^) layer. As no complete removal of the SEI layer was observed when sputtering with 5 keV Ar_1500_^+^ ions, the sputter ion energy and current were increased to 10 keV at 5 nA in order to increase the sputter yield, so that a complete SEI removal is achieved throughout the measurement. With these changed parameters, plateau intensities for Li_2_^−^ and LiH_2_^−^ were observed, indicating a complete removal of the SEI layer and the presence of metallic lithium (Fig. 1b). Comparable to the depth profiles using 5 keV Ar_1500_^+^ sputter ions, maximum intensities for the SI LiCO_3_^−^, C_2_H_3_O^−^ and ^6^LiF_2_^−^ were found at the top surface. These were followed by maximum intensities for LiO^−^, close to the lithium metal surface. As the SEI is reported to be inhomogeneous throughout its depth, featuring an organic outer layer and inorganic inner layer close to the lithium metal, a sufficient separation of these SEI sub-layers is desirable. Indeed, the depth profiles based on Ar_1500_^+^ sputtering (5 keV and 10 keV) exhibited overlaying intensities for the organic (C_2_H_3_O^−^) and the inorganic layer (LiF_2_^−^). In addition to these observations from the ToF-SIMS depth profiles, the sputter-induced surface damage was evaluated via scanning electron microscopy (SEM) analysis. The sputtered surface (10 keV Ar_1500_^+^ sputter ions) appeared irritated and rough compared to the unsputtered surface (Fig. 2a, d). Instead of a homogeneous removal of material, the sputter ion bombardment resulted in the formation of new micrometer-sized structures on the lithium metal surface (Fig. 2d). Probably, these are the result of high total energy input by the polyatomic ToF-SIMS sputter ions, leading to a local melting of the lithium metal surface. Pronounced changes in the surface morphology caused by the sputter process must be avoided, as these lead to mixing of surface layers and changes in the chemical composition upon ToF-SIMS analysis. Since these changes in lithium metal surface morphology were observed for both Ar_1500_^+^ sputter ion energies (see also Fig. S1b), this sputter ion was discarded for further investigations. At this point, the authors would like to point out the limitations of the investigations performed. The influence of methodological phenomena during the sputtering process and SIMS analysis can only be estimated, as they were not further investigated in this study. The measured SI intensity is not only related to the concentration but also to the matrix in which the analyte is embedded. The surrounding matrix influences the ionization probability for each SI, thus possibly leading to an increase or decrease in intensity. The SEI is a very complex and inhomogeneous matrix; therefore, matrix effects can have a significant impact on the measured depth profiles. In addition, preferential sputtering can affect the depth profile. Especially when using gas cluster ion sources, the sputter yield for organic species is higher compared to inorganic species, which can lead to a change in the shape and location of the signals in the measured ToF-SIMS depth profile.

In contrast to the polyatomic Ar_1500_^+^ sputter ions, the ToF-SIMS depth profiles utilizing monoatomic sputter ions (500 eV Cs^+^ and Ar^+^ sputter ions) caused a less sputter-distorted surface in SEM investigations after the measurements (Fig. 2b, c, e, f). The sputtered lithium metal surface was still flat with a grooved structure, originating from the cutting process itself. This grooved structure was also observed for the unsputtered areas (see Fig. S1a). The ion bombardment during ToF-SIMS depth profiling caused the removal of crystalline residues originating from conducting salt, which were present in the unsputtered areas but absent in the sputtered areas. Additionally, the SEM contrast was changed between the unsputtered and sputtered surface, due to the removal of the very top SEI layer exposing the bare lithium metal underneath. (Fig. 2b, c). On the microscopic scale, a sub-micrometer grain formation was observed in the SEM images of the sputtered areas (Fig. 2e, f). Due to the sputter bombardment, the passivating SEI layer was removed and reactive lithium was exposed to traces of atmospheric contaminants in the ToF-SIMS sample chamber, and during SEM analysis. This reaction possibly caused the observed grain formation. In total, both monoatomic sputter ions (Cs^+^ and Ar^+^) featured favored sputter properties in SEM analysis. The SEI layer was removed but no pronounced morphology changes, likely leading to mixing effects, were observed.

Furthermore, the ToF-SIMS sputter depth profiles for both monoatomic sputter ions (Cs^+^ and Ar^+^ sputter ions) were evaluated regarding their capability to examine the layered nature of the SEI. The depth profile utilizing 500 eV Cs^+^ sputter ions at 20 nA showed a rapid (sputter ion fluence <6 × 10^15 ^ions $\cdot$ cm^−2^; 41 s) intensity decrease for all SEI-related SI (Fig. 1c, d). This was followed by an increase of intensity for the bulk-related SI Li_2_^−^ and LiH_2_^−^, indicating the removal of the SEI layer and exposure of the bare lithium metal. In the first section of the depth profile (Fig. 1d), the top SEI layer is clearly separated from the bulk lithium metal. However, the layered nature of the SEI was apparent to a minor extent only. The SI corresponding to carbonate decomposition species (C_2_H_3_O^−^, LiCO_3_^−^ and LiO^−^) featured a comparable depth profile among themselves, with an increase at the beginning of the measurement and an intensity decrease close to the bulk metal. The LiF^−^ intensity increased at a minor rate compared to the carbonate-related SI and the maximum intensity for LiF^−^ was found closer to the bulk lithium metal interface (marked by rising SI intensities for LiH_2_^−^ and Li_2_^−^). The observed SI profiles suggest an organic carbonate-based SEI in the outer layer and an inorganic conducting salt-based layer closer to the lithium metal, which matches common SEI models^11^. Nevertheless, based on the performed measurements, it is not possible to exclude a destruction of molecular species (e.g., C_2_H_3_O^−^) due to ion bombardment. This has the potential to affect data interpretation and is another possible explanation for the observed decrease in intensity for organic species prior to inorganic species. When elucidating the SEI chemistry of unknown samples (e.g., innovative electrolyte formulations), it is recommended that additional measurements are performed with complementary methods. The measured ToF-SIMS depth profiles exhibit a broad overlap of the SI profiles corresponding to the organic and inorganic SEI components and the sputter removal of the SEI took only 41 s (equals 42 SIMS analysis scans). In order to improve the depth resolution and extend measurement time for the SEI, it was necessary to reduce the sputter ion energy. At 250 eV sputter ion energy, the sputter yield was too low to remove surface damage caused by the PI bombardment. This was observed by different contrasts in ToF-SIMS secondary electron images for the sputtered area, bombarded by PI and sputter ions, compared to the outer area, bombarded by sputter ions only (see Fig. S2). Dual source ToF-SIMS sputter depth profiling, as performed in this study, intends a removal of PI-induced surface degradation by the constant bombardment with sputter ions. However, this prerequisite was not met. Possible solutions to enable 250 eV Cs^+^ depth profiling are the increase of sputter ion current or the use of a non-interlaces sputter depth profiling (temporal separation of sputter ion bombardment and SIMS measurement). The first solution was not feasible with the instrumental setup, as the maximum sputter ion current, limited by the ion source, was used already. The second leads to a considerably increased total measurement time. In total, the ToF-SIMS depth profiles utilizing 500 eV Cs^+^ sputter ions featured a rapid removal of the investigated SEI while the layered nature of the SEI was resolved to a minor extent only. At a reduced acceleration voltage of 250 eV, the Cs^+^ sputter ions did not fulfill the requirements of dual source ToF-SIMS sputter depth profiling. Consequently, Cs^+^ sputter ions were dismissed in this study, but further adaption of parameters may be part of future studies.

In the following, the use of monatomic Ar^+^ sputter ions is discussed. Due to the inert characteristics of Ar^+^ sputter ions, we suppose a minor influence on the surface chemistry compared to the electron-donating characteristics of Cs^+^^22,23,34,35^. The ToF-SIMS depth profiles utilizing 500 eV Ar^+^ sputter ions featured a sufficient separation of SEI layer and the bulk material (Fig. 1e). In contrast to the previously discussed depth profiles utilizing Cs^+^ sputter ions, the layered nature of the SEI was resolved in the depth profiles using 500 eV Ar^+^. At low sputter ion fluence, maximum intensities for LiCO_3_^−^, C_2_H_3_O^−^, and high intensities for LiO^−^ were measured. This was followed by an increase of intensity for LiF_2_^−^, corresponding to a layer of conductive salt decomposition products. Closer to the lithium metal the maximum intensity for LiO^−^ was detected, this is possibly due to an instability of lithium carbonate against lithium metal resulting in a decrease of LiCO_3_^−^ and an increase of LiO^−^ intensity closer to the metal surface^42^. Another possible explanation for this increase of LiO^−^ intensity during sputtering, is a sputter-induced decomposition of e.g., lithium carbonate to lithium oxide^43^. With continuous sputter ion bombardment, the bulk lithium metal was reached, as indicated by the maximum values for LiH_2_^−^ and Li_2_^−^. In conclusion, the use of 500 eV Ar^+^ sputter ions was found to be optimal for the ToF-SIMS depth profiling analysis of SEI on lithium metal samples. In the ToF-SIMS sputter depth profiles, the SEI was observed as a separate layer with a layered nature itself. The ToF-SIMS depth profiles matched the expected SEI structure, as described in common SEI models (e.g., by Peled et al.)^11^. The SEM analysis of the sputtered surface showed no pronounced changes in the surface morphology, while successfully removing residues of conducting salt and the SEI during sputter bombardment.

**Fig. 3:**
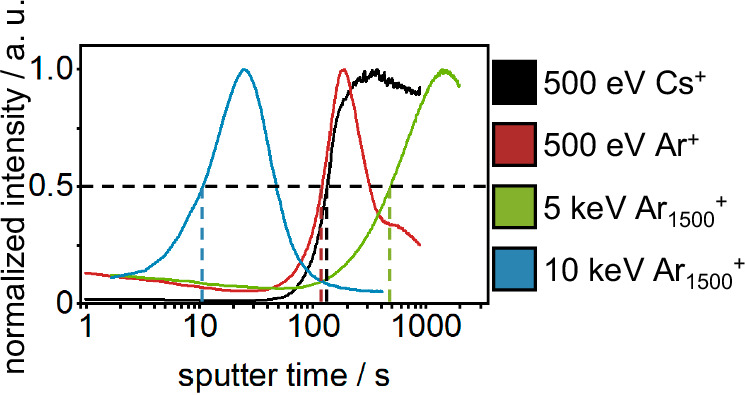
ToF-SIMS sputter depth profiles of a reference material (30 nm Li on Cu). The sputter rates are calculated based on the sputter time at half maximum of Cu^−^, indicated by the dashed lines, and the thickness of the PVD-deposited lithium (30 nm). A similar graph, based on the sputter ion fluence, is given in Fig. S3.

In an attempt to determine the thickness of the analyzed SEI layer, sputter rates were measured on an intended matrix-matched reference material (30 nm lithium metal PVD-deposited on copper foil) for all previously discussed sputter ions. The sputter rates were calculated based on the sputter time at 50% of the maximum intensity of Cu^−^ SI and the thickness (30 nm) of the PVD-deposited lithium (Fig. 3). The calculated sputter rates are (6.04 ± 0.13) × 10^−2^ nm⋅s^−1^ (5 keV Ar_1500_^+^ @ 0.5 nA), (2.69 ± 0.13) nm⋅s^−1^ (10 keV Ar_1500_^+^@5 nA), (2.24 ± 0.06) × 10^−1^ nm⋅s^−1^ (500 eV Cs^+^ @ 20 nA) and (2.46 ± 0.03) × 10^−1^ nm⋅s^−1^ (500 eV Ar^+^ @ 20 nA). These sputter rates for the reference material and the sputter time when reaching the bulk lithium metal of the SEI-covered lithium sections (50% of the maximum Li_2_^−^ intensity) were used to calculate the thickness of the investigated SEI. The calculated SEI thickness ranged vastly from (10 ± 2) nm (Cs^+^), over (52 ± 7) nm (Ar^+^), and (84 ± 38) nm (10 keV Ar_1500_^+^) to >196 nm (end of measurement; 5 keV Ar_1500_^+^). The observed variance of the calculated SEI thickness for the same SEI on lithium metal sample, can only be explained by the different sample matrices of the SEI and the reference material. The PVD-coated sputter reference material featured a metallic lithium layer on metallic copper foil. In contrast to that, the SEI is a matrix of various organic and inorganic components. These different matrices possess different sputter thresholds and the used sputter ions are different in their collision mechanic (monoatomic vs polyatomic). Additionally, Cs^+^ is reported to form a monolayer of cesium on the sample surface, thus, altering the surface chemistry by electron donation to the substrate^22,34,35^. This influence on the surface chemistry is likely different between both samples, due to their different surface chemistries (metallic vs. organic/ionic). Although the reference material was intended to be matrix-matched, as both samples (reference and SEI on lithium metal) are lithium-rich layers, the interaction between the sputter ions and the sample surfaces was observed to be vastly different. This experiment underlines the challenge of depth calibration for thin layers in battery research by a sputter reference material. Especially for measurements of SEI thickness, the development of a matrix-matched sputter standard is a very complex task, due to the high variety of components and layered nature. Instead of reporting a depth by external depth calibration, the illustration of sputter ion fluence should be favored in order to report reproducible results. Although the sputter ion fluence does not give a concrete value for the thickness of the measured layer, it allows for a reliable comparison between measurements with comparable sputter conditions and of similar sample matrices.

### Adaption for thick-layer depth profiling

**Fig. 4:**
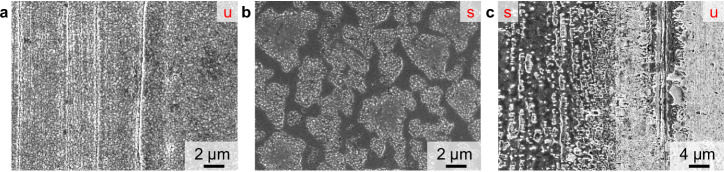
SEM images of lithium metal foil covered by an intermetallic LiZn coating. The unsputtered surface is marked by a red “u” and the sputtered area by a red ‘s’. In **a** the unsputtered LiZn surface coating, in **b** the surface after depth profiling and in **c** an area at the crater edge is given.

The second part of this study relates to the adaption of parameters for monoatomic Ar^+^ sputter depth profiling of thin layers on lithium metal (<100 nm) to be suited for the analysis of thicker surface layers, such as artificial protective coatings (100 nm–2 µm). The sputter ion energy and ion current were increased in order to enhance the sputter yield. As an exemplary application, lithium metal with an intermetallic LiZn coating, as well as corresponding reference samples (lithium metal foil and roll-pressed lithium metal foil) were investigated utilizing 2 keV Ar^+^ sputter ions. Stan et al. first reported the preparation of this protective intermetallic LiZn layer via sputter coating, in a subsequent study Bela et al. reported a PVD preparation method, which was used in this study^44,45^. By SEM analysis, the morphology of the surface was examined in the sputtered and unsputtered areas of the sample after ToF-SIMS depth profiling. The unsputtered coating (Fig. 4a) showed a granular sub-micrometer surface structure originating from the PVD-based coating process. In the sputtered area, the surface morphology was changed considerably to a less granular but more inhomogeneous surface pattern (Fig. 4b, c). The surface was interrupted by unevenly-sized areas with crystalline-like substructures. These structures are expected to be the result of a reaction of reactive lithium with atmospheric contaminations after ToF-SIMS sputter depth profiling. As the protective LiZn coating and lithium passivation layers (e.g., lithium nitride or lithium oxide) are removed during the sputter process, the bare lithium metal is highly prone to react with traces of gaseous contaminants after ToF-SIMS measurement. At the edge of the sputter crater (Fig. 4c), the subsequent removal of material by the sputter ion beam was observable. The granular LiZn coating was gradually removed, as visible from the less bombarded area (right), still featuring LiZn coating, to the crater-near area (left), already exposing the submerged bare lithium metal surface.

**Fig. 5:**
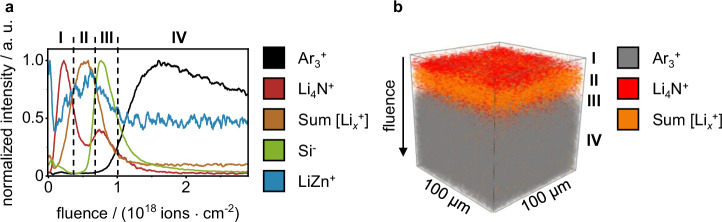
ToF-SIMS sputter depth profiles of lithium metal foil covered by an intermetallic LiZn coating. In **a** the sputter depth profile is given as a graph, the SI intensity is normalized to the respective SI intensity maximum, the fluence corresponds to the sputter ion fluence. **I** to **IV** indicate the assigned layers. The three-dimensional reconstruction of the data is given in **b** (only three SI are given for visibility).

The surface structure of the intermetallic LiZn coating on lithium metal foil was evaluated by ToF-SIMS sputter depth profiling utilizing 2 keV Ar^+^ sputter ions (Fig. 5). In these measurements, a distinct layering of the sample was observed. At low sputter ion fluence, maximum intensities for Li_4_N^+^ were found (marked as **I** in Fig. 5a). These are likely attributed to reaction products of the intermetallic coating with nitrogen residues inside of the glovebox after PVD-processing. This reaction results in the formation of lithium nitride on the PVD-prepared LiZn coating. Below this degradation layer, an unimpaired layer of intermetallic LiZn was present (marked by **II** in Fig. 5a). Surprisingly, only a minor intensity was found for the binary SI LiZn^+^ in this intermetallic layer. However, Li_*x*_^+^ (*x* = 4–9) SI was present in this layer and collocated with the measured intensity of LiZn^+^. As these Li_*x*_^+^ SI were collocated with the intensity for LiZn^+^ and absent in the bulk lithium metal (marked by **IV** in Fig. 5a), they were supposed as characteristic SI for the intermetallic LiZn phase. The low intensity for LiZn^+^ in the prepared LiZn coating may be related to the low electronegativity of lithium resulting in a preferred ionization of lithium cluster SI and a suppression of Zn-containing SI. Based on the measured data, it is not possible to make a definitive statement on the ionization processes that lead to the observation of Li_*x*_^+^ SI in the intermetallic coating, while observing very minor intensity for Li_*x*_^+^ in the bulk material during SIMS analysis. Consequently, this hypothesis remains tentative and is not further explored in this study. Further sputter ion bombardment revealed the presence of a second intensity increase for Li_4_N^+^ underneath the intermetallic layer, likely corresponding to a second lithium nitride layer (marked by **III** in Fig. 5a). Interestingly, this Li_4_N^+^ maximum was collocated in the depth profile with the maximum intensity of Si^−^. This information on collocation was obtained by combining the depth profiles in positive and negative SIMS polarity under identical sputter conditions. The overlay of datasets in both polarities is possible for this sample due to a uniform PVD coating. To confirm uniformity, multiple measurements were performed at various locations of the PVD-coated sample, exhibiting comparable depth profiles. The same collocation of Li_4_N^+^ and Si^−^ was observed for the measured reference samples (pristine lithium foil and roll-pressed lithium metal foil). For both reference samples, a layer of Li_4_N^+^ and Si^−^ was present in the depth profiles at low sputter ion fluence, immediately decreasing in intensity with increasing sputter ion fluence (see Fig. S4a, b). Probably, this Li_4_N^+^-containing surface layer is the product of a reaction during lithium metal foil-manufacturing. During the foil manufacturing and roll-pressing process, lithium metal is mechanically formed to the desired thickness, which results in the exposure of reactive lithium metal to the surrounding atmosphere. The main constituent of ambient atmosphere (e.g., in a dryroom) is nitrogen and gloveboxes without purification system have an unmonitored nitrogen contamination. Consequently, the exposed reactive lithium metal reacts to lithium nitride, a reactivity that has been studied in literature^46,47^. The presence of Si^−^ in this layer is likely due to contamination with e.g., silicone oils during manufacturing. Performing the roll-pressing process resulted in a higher Si^−^ intensity (see Fig. S4c) compared to the pristine lithium foil, due to the additional contact to the silicone-coated polymer foil. This increased Si^−^ intensity is an indication of increased surface contamination with silicon-contaminants after roll-pressing. However, as SI intensity in SIMS is not only linked to analyte concentration but also matrix effects, advanced investigations would be necessary to further support this thesis. Since this layer of colocalized Li_4_N^+^ and Si^−^ was observed in the depth profiles of the lithium metal substrate (roll-pressed lithium metal, see Fig. S4a, b) as well as for the PVD-coated sample, it was assigned to the native passivation layer of the roll-pressed lithium metal foil substrate.

Underneath this native passivation layer of the lithium metal substrate, a rise of intensity for Ar_*x*_^+^ SI was observed in the ToF-SIMS depth profile (marked by **IV** in Fig. 5a). These Ar_*x*_^+^ SI were also observed during the sputter depth profiling of both reference samples (pristine and roll-pressed metal lithium foil). For these reference samples, the increase of Ar_*x*_^+^ was observed after sputter removal of the native passivation layer, marked by the decrease of Li_4_N^+^ and Si^−^ intensity. Since the reference samples consist of bulk lithium underneath the native passivation layer, the presence of Ar_*x*_^+^ SI was attributed to the presence of bulk lithium metal. Other distinct lithium-containing SIs for the presence of bulk lithium metal were not found throughout the depth of the bulk material. After reaching a maximum intensity for Ar_3_^+^ by implantation of argon in the lithium metal surface, the intensity decreased at a low rate, possibly due to successive surface damage caused by the ion bombardment.

In total, four layers were identified on the PVD-coated sample. The very top layer was a Li_3_N-containing degradation layer of the prepared LiZn coating (**I**). Underneath, the unimpaired LiZn-phase was found (**II**). Further sputtering exposed the native passivation layer of the lithium metal (**III**), comprising N- and Si-containing components, probably Li_3_N and silicone oils. Reference samples indicated that the Si-containing components partly originated from contact with siliconized foil during the roll-pressing process. Lastly, the bulk lithium metal was exposed (**IV**), which initially served as a substrate for the PVD coating. A three-dimensional reconstruction of the depth profile is given in Fig. 5b as well as a simplified schematic representation of the PVD-prepared LiZn coating in Figure S5.

## Conclusions

In this study, a comparison of ToF-SIMS sputter ions (Cs^+^, Ar^+^, and Ar_1500_^+^) as well as suited parameters for the analysis of the SEI layer on lithium metal are reported. Based on the depth profiles and the SEM analysis after sputter depth profiling, Ar_1500_^+^ sputter ions (5 and 10 keV) were discarded for the given investigations. The corresponding depth profiles missed the expected layering of the SEI, additionally, the surface morphology was strongly altered due to the ion bombardment. A successful depth profiling of the SEI was achieved by both monoatomic sputter techniques (Cs^+^ and Ar^+^ sputter ions). Nevertheless, to improve depth resolution for Cs^+^-sputtering the sputter energy must be reduced further. At a reduced ion energy of 250 eV an incomplete removal of PI-induced surface damage by the sputter ions was observed. ToF-SIMS depth profiling utilizing 500 eV Ar^+^ exhibited a good separation of the SEI layers and the bulk lithium metal in a reasonable measurement time. The organic and inorganic layers of the SEI were well resolved in the depth profile. The observed SEI layering was in accordance with the common SEI model of Peled et al.^11^. Apart from the changed SEM contrast, the morphology of the lithium metal surface after sputtering was still comparable to the unsputtered lithium metal surface. In an attempt to determine the SEI thickness, a reference material was applied for external thickness calibration. However, these investigations using a reference material, which was intended to be matrix-matched, underlined the challenge of external depth calibration for complex matrices such as the SEI.

For the analysis of thicker surface layers (100 nm to 2 µm), the sputter parameters were adapted by increasing the sputter ion energy and current. As an exemplary system, lithium metal covered by an intermetallic LiZn coating was investigated by ToF-SIMS depth profiling. The layered nature of this model system was revealed, including a Li_3_N-containing degradation layer of the coating, the prepared intermetallic LiZn-phase, Si- and Li_3_N-containing native passivation layer, and the underlying lithium metal foil substrate. The overlay of datasets in positive and negative SIMS polarity allowed for a comprehensive evaluation of the coating structure. For both, the intermetallic phase and the bulk material, characteristic SI was assigned.

In conclusion, the comparison of different sputter ions performed in this study assists in the selection of appropriate measurement conditions for future investigations of lithium metal surfaces. The optimum sputter conditions (sputter ions and parameter) for the investigated thin-layer (SEI on lithium metal sections) and thick-layer (intermetallic LiZn coating on lithium metal foil) samples were based on 500 eV (for thin-layers) and 2 keV (for thick-layers) Ar^+^ sputter ions. In both applications, ToF-SIMS sputter depth profiling provided a comprehensive insight into the layer structure and surface chemistry. These applications underline the value of ToF-SIMS sputter depth profiling as a powerful tool for understanding and improving lithium metal | electrolyte interfaces and interphases in battery research.

## Methods

### Lithium metal sections covered with SEI

Lithium metal sections applied for comparison of the sputter ions were prepared by cutting a lithium metal rod (Sigma-Aldrich) submerged in the electrolyte (1.2 M LiPF_6_ in ethylene carbonate/ethyl methyl carbonate (3/7 by weight); E-lyte Innovations GmbH) with a tungsten wire. The procedure is described in more detail in a previous publication^41^.

### Roll-pressed and PVD-coated lithium metal samples

500 µm lithium metal (Honjo Metal Co., Ltd.) was roll-pressed between two siliconized polyester foils (50 µm, PPI Adhesive Products GmbH) to a thickness of 130 µm using an GK300L roll-press calender (Saueressig Group) in a dry room atmosphere (dew point <−60 °C). For further PVD coating the samples were transferred in an Ar-filled glovebox (M. Braun Inertgas-Systeme GmbH; H_2_O ≤ 0.1 ppm, O_2_ ≤ 0.1 ppm) using a ProVap PVD system (M. Braun Inertgas-Systeme GmbH). The PVD coating procedure is described in more detail in a previous publication^45^.

The sputter reference sample was prepared by coating lithium metal on a copper foil via an adaption of the previously mentioned PVD coating method^45^.

### Time of flight—secondary ion mass spectrometry

ToF-SIMS measurements were carried out on a TOF.SIMS 5 (Iontof GmbH). The instrument was equipped with a 30 kV bismuth liquid metal primary ion source, a 10 kV gas cluster ion source, a 2 kV cesium ion source, and a 2 kV electron impact ion source. 30 keV Bi_3_^+^ ions were chosen as primary ions, operated with a PI current of 2.0–2.2 pA (Cs^+^ or Ar^+^ as sputter ion) and 1.3 pA (for Ar_1500_^+^ as sputter ion). The primary ion beam was rastered randomly in a field of 100 × 100 µm^2^ with 128 × 128 pixels. Charge compensation with low energy electrons was not applied, as no surface charging phenomena were observed. The extraction voltage was set to 2 kV. The time of flight analyzer was operated at a cycle time of 60 µs (100 µs for Ar_1500_^+^ measurements) resulting in a mass range of $\frac{m}{z}$ 1–320. The achieved mass resolution was minimum $\frac{m}{\Delta m}$ 6000 (FWHM at $\frac{m}{z}$ 14.03 (Li_2_^−^)). SI assignment was performed based on $\frac{m}{z}$, isotopes and chemical identity of the sample. The gas cluster source was operated with argon gas (purity 99.999%; Westfalen AG) at 10 kV acceleration voltage and an ion current of 5 nA, respectively, at 5 kV and an ion current of 0.5 nA. The cesium source was used at an ion energy of 500 eV and 20 nA ion current. The electron impact sputter ion source was operated with argon gas (purity 99.999%; Westfalen AG) at 500 eV sputter ion energy and 20 nA ion current. All sputter ion sources were rastered in a field of 300 × 300 µm^2^, in order to avoid crater edge effects during SIMS analysis. The interlaced sputter mode was used. The ion sources were tuned daily with respect to reproducible ion currents at the sample stage and beam focus in order to ensure reproducible measurement conditions. Additionally, the gas cluster ion source was tuned with respect to the main size of the argon cluster distribution (e.g., Ar_1500_^+^ corresponds to the main cluster size but includes smaller/bigger clusters). The measured main argon cluster size for each measurement was Ar_1500±50_. Prior to analysis, the samples were handled in an Ar-filled glovebox (H_2_O ≤ 0.1 ppm, O_2_ ≤ 0.1 ppm) only. Within this inert Ar-atmosphere, the samples were mounted conductively in a backmount sample holder (Iontof GmbH) and placed in an Ar-filled transfer vessel (Iontof GmbH). The transfer vessel was used for transport of the samples from the glovebox in the instrument. Data evaluation was carried out using the SurfaceLab 7.3 software pack (Iontof GmbH). In the depicted depth profiles, each datapoint corresponds to one analysis scan, the profiles were smoothed by a running average algorithm (*n* = 10).

### Scanning electron microscopy

Scanning electron microscopy (SEM) analysis was performed using a CrossBeam 550 working station (Carl-Zeiss AG) equipped with a field emission gun working at an acceleration voltage of 3 kV and a working distance of 5.1 mm. After the ToF-SIMS experiments, the lithium metal samples were transferred from the ToF-SIMS instrument to an Ar-filled glovebox (M. Braun Inertgas-Systeme GmbH; H_2_O ≤ 0.1 ppm, O_2_ ≤ 0.1 ppm) using the ToF-SIMS transfer vessel. The samples were placed in the air-tight SEM sample holder and transferred to the microscope.

## Supplementary information

Supporting Information

## Data Availability

The authors declare that the data supporting the findings of this study are available within the paper and its Supplementary Information files. Should any raw data files be needed they are available from the corresponding author upon reasonable request.
