# Supplementary material for: ToF-SIMS sputter depth profiling of interphases and coatings on lithium metal surfaces
Source: Commun Chem. 2025 Feb 3;8:31. doi: 10.1038/s42004-025-01426-0 (PMC11790834; doi:10.1038/s42004-025-01426-0)
Supplement: Supplementary file 1 — Supporting Information [file 42004_2025_1426_MOESM1_ESM.pdf]

Supporting Information

ToF-SIMS Sputter Depth Profiling of Interphases and Coatings on Lithium Metal Surfaces

Maximilian Mense[1], Marlena M. Bela[1], Sebastian P. Kühn[2], Isidora Cekic-Laskovic[2], Markus Börner[1], Simon Wiemers-Meyer[1], Martin Winter[1,2], Sascha Nowak[1*]

1 University of Münster, Münster Electrochemical Energy Technology (MEET), Corrensstraße 46, 48149 Münster, Germany

2 Helmholtz-Institute Münster, IMD-4, Forschungszentrum Jülich GmbH, Corrensstraße 46, 48149 Münster, Germany

*corresponding author: sascha.nowak@uni-muenster.de

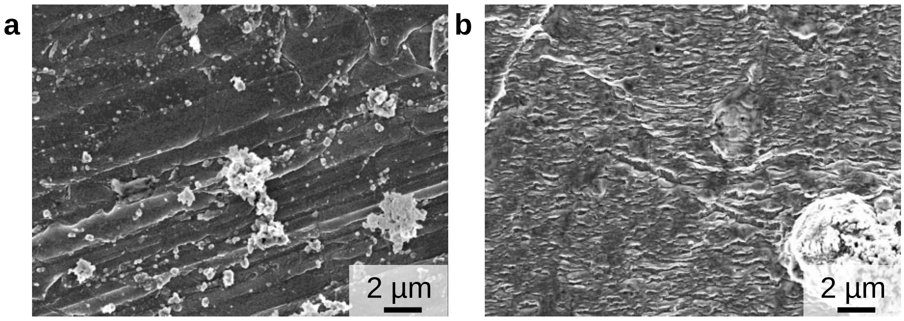

Fig. S1 | SEM images of the lithium metal surface cut under electrolyte. In **a**, the unsputtered sample surface of the SEI-covered lithium is shown. **b** shows the surface after sputter depth profiling with 5 keV Ar_{1500}^{+} at 0.5 nA. Both images were taken at 5k magnification.

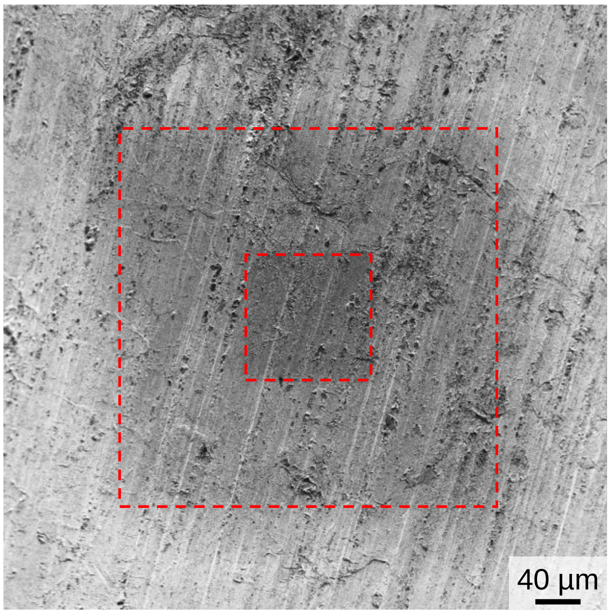

Fig. S2 | Secondary electron image from ToF-SIMS. Ga^{+} PI ions were used, image was taken after ToF-SIMS sputter depth profiling with 250 eV Cs^{+} as sputter ions and 30 keV Bi_3^{+} as PI. The dashed lines mark the affected areas by sputter bombardment only (outer square) and by sputter ions and PI bombardment (inner square).

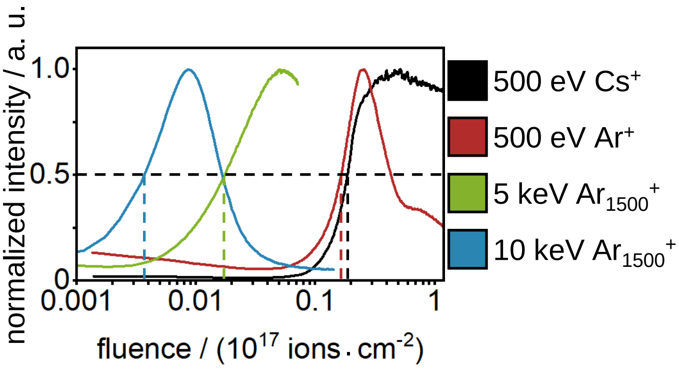

Fig. S3 | ToF-SIMS sputter depth profiles of a reference material (30 nm Li@Cu). Similar data to Figure 3 with a changed x-axis to sputter ion fluence is depicted. Dashed lines at 50 % of the maximum Cu^{-} intensity. The thickness of the PVD-deposited lithium is 30 nm.

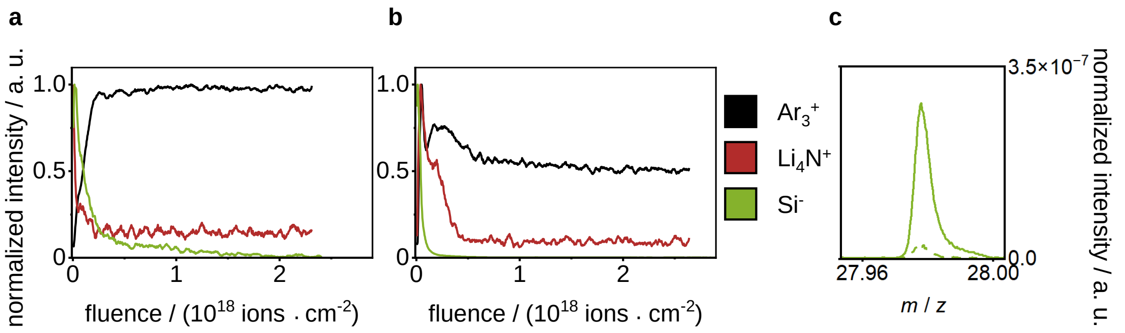

Fig. S4 | ToF-SIMS depth profiling data of the reference samples. The given fluence corresponds to the sputter ion fluence. In **a**, the ToF-SIMS depth profile of pristine Li foil is shown, **b** displays the depth profile of roll-pressed Li metal. **c** gives a comparison for the Si signal of both reference samples, the SI intensity is normalized to the total PI dose, the dashed line represents lithium foil, the solid line the roll-pressed reference.

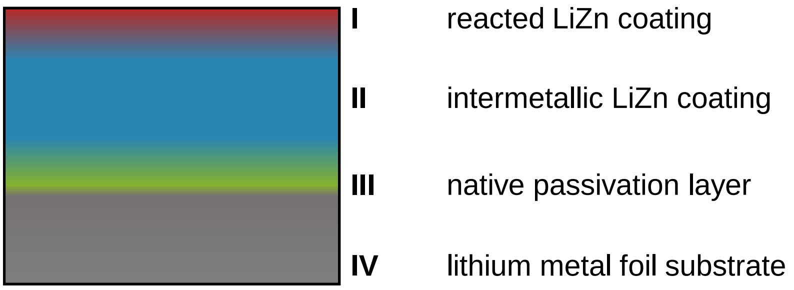

Fig. S5 | Schematic representation of the layered structure of the intermetallic LiZn coating on the lithium metal foil substrate prepared by PVD. The scheme is based on the experimental observations in ToF-SIMS depth profiling.
